# Supplementary material for: Impacts of Harvest Year and Cultivation Location on Off-Flavor Compounds and Functionality of Pea Protein Isolate
Source: Foods. 2024 Oct 27;13(21):3423. doi: 10.3390/foods13213423 (PMC11545078; doi:10.3390/foods13213423)
Supplement: Supplementary file 1 [file foods-13-03423-s001.zip › foods-3260423-supplementary.pdf]

SUPPLEMENTARY

Table S1: Proximate compositions of pea flours and protein isolates from different harvesting years and cultivation locations.

	Dehulling [%]	Grinding [%]	Ash [%]		Moisture [%]		Fat Content [%]	
			Flours	Protein Isolates	Flours	Protein Isolates	Flours	Protein Isolates
Harvest year								
2018	83.3 ± 1.2^{a}	98.7 ± 0.3^{a}	1.97± 0.1^{a}	4.22 ± 0.4^{a}	8.18 ± 0.6^{a}	1.46 ± 0.1^{a}	6.00 ± 1.4^{a}	12.46 ± 0.7^{a}
2019	84.9 ± 0.4^{a}	99.0 ± 0.0^{a}	2.04 ± 0.2^{a}	6.10 ± 0.5^{b}	8.33 ± 0.3^{a}	1.86 ± 0.1^{b}	5.85 ± 0.001^{a}	13.01 ± 1.0^{a}
2020	84.2 ± 0.9^{a}	98.8 ± 0.0^{a}	2.91 ± 0.01^{c}	6.38 ± 0.7^{b}	8.98 ± 0.4^{a}	1.65 ± 0.1^{ab}	3.57 ± 0.03^{a}	12.86 ± 0.2^{a}
2022	86.6 ± 0.5^{a}	98.6 ± 0.0^{a}	2.59 ± 0.05^{b}	6.26 ± 0.2^{b}	11.00 ± 0.7^{b}	1.92 ± 0.2^{b}	4.79 ± 0.2^{a}	12.81 ± 0.4^{a}
Cultivation location								
1	85.1 ± 1.9^{a}	99.2 ± 0.1^{a}	2.74 ± 0.01^{a}	6.36 ± 0.04^{b}	9.42 ± 0.2^{a}	2.45 ± 0.1^{a}	3.47 ± 0.03^{a}	12.34 ± 0.4^{a}
2	84.7 ± 0.4^{a}	99.1 ± 0.1^{a}	2.33 ± 0.02^{b}	4.76 ± 0.05^{a}	10.37 ± 0.2^{b}	2.32 ± 0.2^{a}	3.08 ± 0.2^{a}	12.41 ± 3.1^{a}

Results are expressed as means ± standard deviation (n =2 or 3). $^{a-c}$ Column values followed by the same letter are not significantly different ($p > 0.05$).

Table S2: Color and yellowness/whiteness of pea flours and protein isolates from different harvesting years and cultivation locations.

Flours						Protein Isolates				
	L	a	b	Yellowness	Whiteness	L	a	b	Yellowness	Whiteness
Harvest years										
2018	90.5 ± 0.1	0.15 ± 0.04	22.7 ± 0.3	35.82 ± 0.5^{d}	75.40 ± 0.3^{a}	82.82 ± 0.3	2.4 ± 0.13	28.53 ± 0.7	49.21 ± 1.3^{c}	68.42 ± 0.3^{ab}
2019	90.8 ± 0.03	-0.63 ± 0.02	20.51 ± 0.2	32.29 ± 0.3^{a}	77.50 ± 0.1^{d}	82.11 ± 0.23	1.55 ± 0.2	24.2 ± 0.22	42.06 ± 0.5^{a}	69.89 ± 0.3^{c}
2020	90.7 ± 0.04	-1.54 ± 0.04	21.21 ± 0.2	33.42 ± 0.3^{b}	76.83 ± 0.2^{c}	81.8 ± 0.5	1.35 ± 0.2	26.2 ± 0.2	45.71 ± 0.6^{b}	68.09 ± 0.4^{a}
2022	90.8 ± 0.1	2.38 ± 0.04	22.1 ± 0.13	34.70 ± 0.2^{c}	76.10 ± 0.1^{b}	83.26 ± 0.3	0.79 ± 0.2	26.3 ± 0.4	45.12 ± 0.7^{b}	68.81 ± 0.4^{b}
Cultivation location										
1	91.35 ± 0.2	-0.31 ± 0.04	19.81 ± 0.2	30.99 ± 0.2^{a}	78.38 ± 0.2^{b}	81.46 ± 0.4	0.76 ± 0.09	24.99 ± 0.3	43.82 ± 0.6^{b}	68.88 ± 0.3^{a}
2	90.96 ± 0.1	-0.44 ± 0.03	20.37 ± 0.2	31.99 ± 0.4^{b}	77.71 ± 0.2^{a}	81.46 ± 0.3	0.45 ± 0.06	23.59 ± 0.5	41.37 ± 0.9^{a}	69.99 ± 0.5^{b}

The letters L* a* and b* represent the lightness, green-red and blue-yellow respectively. Results are presented as means ± standard deviation (n=5). Different letters ($^{a-c}$) indicate significant differences ($p > 0.05$).

Table S3, Weather condition across the cultivation year of 2022 for the two studied cultivation locations.

Month	Temperature-Max (°C)		Temperature-Min (°C)		Temperature-Mean (°C)		Humidity--Max (%)		Humidity-Min (%)		Precipitation Daily (mm)		Precipitation since the begining of the year (mm)		Wind (s/s)		Sun beam (h)	
	Location 1	Location 2	Location 1	Location 2	Location 1	Location 2	Location 1	Location 2	Location 1	Location 2	Location 1	Location 2	Location 1	Location 2	Location 1	Location 2	Location 1	Location 2
Jan	3.0	0.6	-3.4	-16.8	8.8	8.0	91.6	86.9	36.0	47.5	73.0	16.2	73.0	16.2	2.5	4.4	55.1	29.9
Feb	3.3	0.7	-3.0	-9.1	9.1	6.6	93.8	87.9	47.1	52.9	122.4	31.0	195.4	47.2	3.0	4.2	111.6	69.9
Mar	3.6	2.0	-10.2	-11.9	39.1	14.6	77.5	72.2	19.7	24.4	1.4	0.0	196.8	47.2	2.2	2.4	356.9	195.0
Apr	6.7	4.5	-7.4	-10.4	41.0	16.9	73.7	69.6	25.2	25.7	62.0	27.0	258.8	74.2	2.5	3.0	471.1	256.6
May	12.0	10.3	-5.8	-4.4	47.1	21.5	80.9	70.5	25.6	25.2	86.2	37.0	345.0	111.2	1.7	2.9	546.4	366.0
Jun	16.8	15.6	-6.3	6.1	64.1	30.8	85.5	77.1	42.3	38.0	42.2	26.8	387.2	138.0	1.3	2.3	584.8	387.8
Jul	23.8	16.3	-1.4	5.1	88.5	35.9	86.9	76.6	32.7	29.5	98.4	55.2	485.6	193.2	1.3	2.2	579.1	390.0
Aug	22.2	17.7	-37.6	5.2	85.6	31.4	78.7	76.1	30.9	28.0	30.4	94.6	516.0	287.8	1.3	2.5	507.9	433.0
Sep	12.9	11.2	1.7	0.9	21.6	19.1	80.0	82.7	37.2	44.0	59.4	33.7	575.4	321.5	1.8	2.7	312.4	224.4
Oct	11.6	9.5	-0.4	-0.6	18.1	16.4	90.8	92.8	51.2	58.8	48.4	56.8	623.8	378.3	1.8	3.1	162.5	84.2
Nov	6.9	4.8	-4.2	-4.5	14.4	14.3	93.5	96.8	70.7	69.0	25.8	20.6	649.6	398.9	2.8	3.1	56.9	31.6
Dec	-0.3	-2.2	-14.3	-16.9	8.5	6.4	96.9	94.1	76.0	77.6	72.4	18.0	722.0	416.9	2.1	2.9	35.3	24.6

Table S4, Soil composition of the two studied cultivation locations.

	Clay content (%)	pH	Phosphorus (%)	Potassium (%)	Potassium/magnesium	Calcium (ppm)
Location 1	25	8	12.3	10	1.05	430
Location 2	38	6,5	7.5	15	1.1	270

Table S5: Emulsion stability and activity index for protein isolates recovered from peas of different harvesting years and cultivation locations.

	EAI	ES
Harvesting Year samples		
2018	26.1 ± 3.1^{b}	12.17 ± 4.4^{a}
2019	20.13 ± 0.5^{ab}	14.81 ± 4.6^{a}
2020	23.32 ± 1.4^{ab}	14.44 ± 4.0^{a}
2022	19.06 ± 0.1^{a}	12.95 ± 0.004^{a}
Cultivation location samples		
1	18.58 ± 3.0^{a}	14.9 ± 3.4^{a}
2	20.77 ± 0.8^{a}	14.2 ± 2.1^{a}

Results are presented as means ± standard deviation (n=2). Different letters ($^{a\text{-}c}$) indicate significant differences ($p > 0.05$).

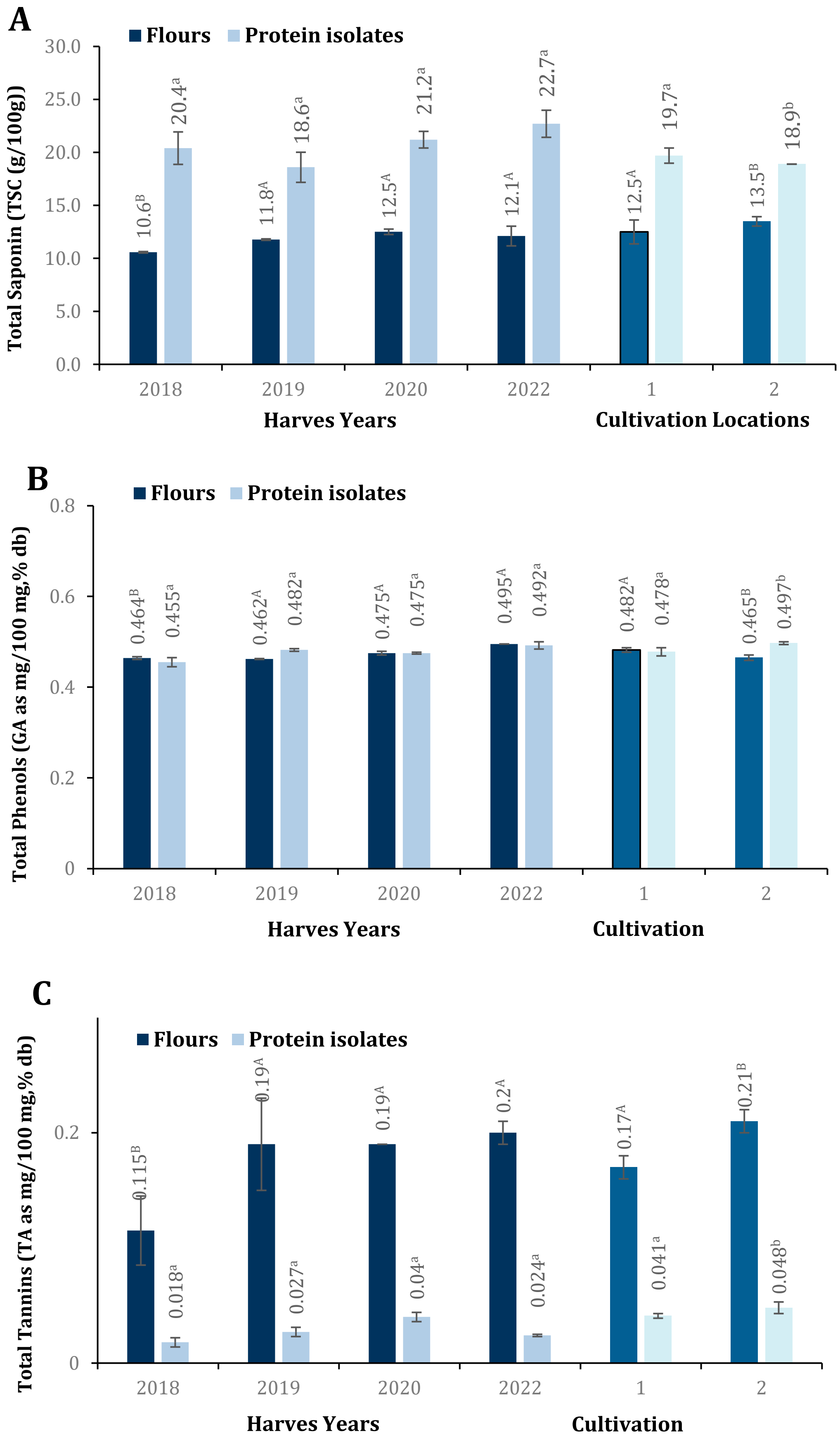

Figure S1: Non-volatile contents in pea flours and protein isolates from different harvesting years and cultivation locations. (A) Total saponin expressed as g of total saponins per 100 g extract; (B) Total

phenols are expressed as mg Gallic Acid Equivalent (GAE) in 100 g of the sample on dry basis (C) Total tannins are recorded as mg Tannic Acid Equivalent (TAE) in 100 g of the sample on dry basis. Values are mean ± SD, ($n = 2$). Values followed by the same letter in the same assay are not significantly different ($p < 0.05$).
